# Adrenal Medullary Hyperplasia: A Systematic Review and Meta-analysis

**DOI:** 10.1210/clinem/dgad121

**Published:** 2023-03-10

**Authors:** Rafal Ganni, David J Torpy, Henrik Falhammar, R Louise Rushworth

**Affiliations:** School of Medicine, Sydney, The University of Notre Dame, Darlinghurst, NSW 2010, Australia; Endocrine and Metabolic Unit, Royal Adelaide Hospital, Adelaide, SA, Australia; Faculty of Medicine, University of Adelaide, Adelaide, SA, Australia; Department of Endocrinology, Karolinska University Hospital, SE-17176 Stockholm, Sweden; Department of Molecular Medicine and Surgery, Karolinska Institutet, SE-17176 Stockholm, Sweden; School of Medicine, Sydney, The University of Notre Dame, Darlinghurst, NSW 2010, Australia

**Keywords:** genetic syndrome, catecholamine excess, adrenalectomy

## Abstract

**Context:**

Adrenal medullary hyperplasia (AMH) is a rare, incompletely described disorder of the adrenal medulla that is associated with catecholamine excess.

**Objective:**

To increase knowledge about AMH by reviewing the reported cases of this disorder.

**Design:**

Systematic review and meta-analysis of the genotype/phenotype relationship in all reported cases of AMH.

**Setting:**

Literature review and analysis.

**Patients or Other Participants:**

All cases of AMH published to date.

**Main Outcome Measure(s):**

Characteristics of AMH cases and genotype-phenotype relationships.

**Results:**

A total of 66 patients, median age of 48 years, were identified from 29 reports. More than one-half were male (n = 39, 59%). The majority had unilateral (73%, n = 48) disease; 71% (n = 47) were sporadic and 23% (n = 15) were associated with the MEN2. Most (91%, n = 60) displayed signs and symptoms of excess catecholamine secretion, particularly hypertension. Elevated catecholamine concentrations (86%, n = 57) and adrenal abnormalities on imaging were common (80%, n = 53). More than one-half (58%, n = 38) had concurrent tumors: pheochromocytoma (42%, n = 16/38); medullary thyroid cancer (24%, n = 9/38); and adrenocortical adenoma (29%, n = 11/38). Most (88%, n = 58) underwent adrenalectomy with 45/58 achieving symptom resolution. Adrenalectomy was less common in patients under 40 years and those with bilateral disease (both *P* < .05).

**Conclusion:**

AMH may be sporadic or associated with MEN2, most have catecholamine excess and imaging abnormalities. Unilateral involvement is more common. Most reported patients have been treated with adrenalectomy, which is usually curative with regard to catecholamine hypersecretion.

Adrenal medullary hyperplasia (AMH) is a rare benign condition of the adrenal gland, the epidemiology of which is poorly described (1). AMH is characterized by the presence of diffuse or nodular radiologically detectable lesions in the adrenal medulla, with episodic or persistent signs and symptoms of catecholamine excess. Clinical features include hypertension, sweating, palpitations, headache, and anxiety (2, 3). AMH can be sporadic or familial, resulting from germline mutations of genes known to cause pheochromocytoma, such as the *RET* gene in multiple endocrine neoplasia type 2 (MEN2) syndrome, or in the *NF1, SDHB,* and *MAX* genes (4). Although clinical and biochemical findings of excess catecholamines (urine or blood) and imaging such as ultrasound, computed tomography, magnetic resonance imaging, and meta-iodobenzylguanidine (MIBG) scanning may help detect an expansion in the adrenal medulla, histological examination revealing a decrease in corticomedullary thickness ratio (<10:1) is diagnostic for AMH (5). AMH bears a symptomatologic resemblance to pheochromocytoma, a benign or malignant tumor of the adrenal medulla characterized also by proliferation of medullary cells with an atypical polygonal shape as opposed to normal cellular architecture seen in AMH. Thus, pheochromocytoma is the main differential diagnosis for AMH. AMH is considered a precursor for pheochromocytoma in patients with MEN2 (4). Catecholamine α- and β-adrenergic receptor blockers are used to ameliorate the clinical features of catecholamine excess in AMH. However, surgical removal of the adrenal gland is the preferred treatment (5) to control catecholamine excess and prevent progression to pheochromocytoma, particularly in patients with MEN2 (4).

Information on the epidemiology, presentation, diagnosis, and outcome of AMH is scarce and based only on case reports or limited case series. In this study, we aim to establish a better understanding of AMH by conducting a thorough systematic review and meta-analysis of all cases of AMH reported to date.

## Methods

### Case Identification

**Figure 1.**
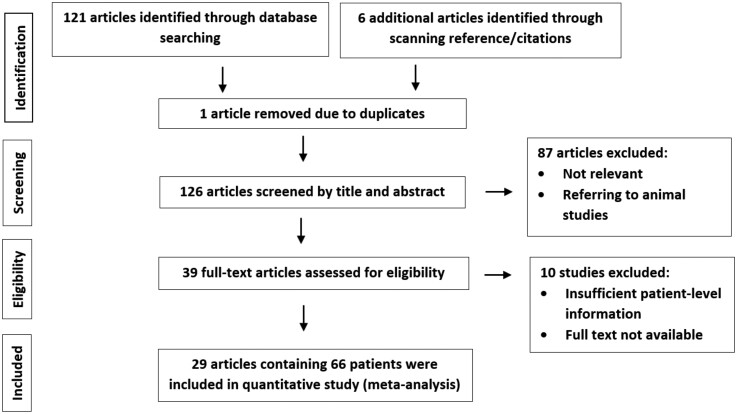
PRISMA flow diagram of the study selection process.

AMH case reports and case series were identified using PubMed and Google Scholar searches conducted up to June 13, 2022. The following keywords were used for both databases: “Adrenal medullary hyperplasia” OR “Adrenomedullary hyperplasia” using “All Fields” and “Show Index” options on PubMed. Following the Preferred Reporting Items for Systematic Reviews and Meta-Analyses guidelines as shown in Fig. 1 (6), 121 articles were identified, with a further 6 articles located from scanning reference and citation lists. One article was excluded because of duplication. By scanning titles and abstracts, articles referring to work conducted on animals or considered irrelevant were excluded. Thirty-nine articles remained after exclusion. These were scanned for eligibility. Of the 39 articles, 10 were excluded because of insufficient patient-level information and full-text unavailability. At the end of this process, 66 patients with AMH were identified from 29 published articles.

Because all data used in this analysis were derived from published case studies, no ethical clearance was required.

### Data Extraction

For each of the 29 articles, the full text was reviewed, and individual patient data were collected and organized into a dataset. Specifically, data were collected on age; nationality; sex; presence of genetic mutations; other concurrent tumors (according to type, including adrenal cortical adenomas); clinical features of catecholamine excess such as elevated blood pressure, headache, palpitations, sweating, and anxiety; urinary and serum catecholamine levels, histology; imaging results; treatments; and outcomes.

Systolic blood pressure (BP) values above 140 mm Hg and/or diastolic BP of 90 mm Hg or more were classified as hypertensive. Where more than 1 BP measurement was given, the average of these was used in the analysis. Paroxysmal hypertension was also recorded, if reported. Where BP measurements (or “hypertension”) were not reported, it was assumed that the patient did not have hypertension for the purposes of this analysis. Urinary or serum total catecholamine, adrenaline, noradrenaline, dopamine, and catecholamine metabolites (metanephrine, normetanephrine, 3-MT, or vanillylmandelic acid) concentrations were extracted. For each of these, any level above the reference range provided in each case study was classified as elevated.

### Data Analysis

Patient data were analyzed according to demographic variables and according to the presence of features associated with AMH. For this analysis, patient age was grouped into “0 to 39 years,” “40 to 59 years,” and “60 years and over” categories. χ^2^ or Fisher exact tests were used to assess the significance of differences in the distribution of categorical variables and *t* tests were used to assess the differences in continuous variables, with adjustment for differences in variance, where appropriate. A *P* value <.05 was used to determine statistical significance.

The data analysis was completed using SPSS (IBM Corp. Released 2020. IBM SPSS Statistics for Windows, Version 27.0. Armonk, NY: IBM Corp).

## Results

Of all AMH cases, 91% (60/66) were confirmed by histology, whereas the remaining cases were categorized as AMH on the basis of imaging and clinical presentation. Of the 66 unique patients in the study, 45 were identified from larger studies (4, 7-13) and the remaining 21 were identified from individual case reports (Table 1) (1, 3, 27-29). Twelve countries were represented in the sample, with Sweden (n = 20, 30%), United States (n = 14, 21%), Japan (n = 9, 14%), and Spain (n = 7, 11%) being the most common.

**Table 1.** Articles assessed for eligibility

Authors	Number of patients	Included	Reason for exclusion
Ansari et al (14)	1	✓	
Bailey et al (15)	1		Full text not available
Bergman et al (16)	1	✓	
Borrero et al (17)	1	✓	
Carney et al (18)	1		Full text not available
Cheng et al (13)	3	✓	
DeLellis et al (8)	3	✓	
Dralle et al (11)	4	✓	
Dupont et al (19)	1		AMH diagnosis not confirmed
Ekelund, Hoevels (20)	1	✓	
Falhammar et al (4)	19	✓	
Feldman et al (21)	1		Insufficient patient-level information
Grogan et al (22)	1	✓	Grogan, Pacak, Pasche, Huynh, Greco (22)
Gupta et al (3)	1	✓	
Huang et al (23)	7		Insufficient patient-level information
Inaba et al (12)	3	✓	
Ishay et al (24)	1	✓	
Jansson et al (25)	12		Insufficient patient-level information
Kawano et al (26)	1	✓	
Kazama et al (27)	1	✓	
Krubsack et al (28)	1	✓	
Kura et al (29)	1	✓	
Letizia et al (30)	1	✓	
Marín et al (7)	7	✓	
Mejia et al (2)	1		Full text not available
Montebello et al (1)	1	✓	
Nishimura et al (31)	1	✓	
Qupty et al (10)	2	✓	
Radin et al (32)	1	✓	
Romanet et al (33)	1	✓	
Rudy et al (9)	4	✓	
Stenger et al (34)	1		Full text not available
Sun (35)	6		Full text not available
Visser, Axt (36)	1	✓	
Yamamoto et al (37)	1	✓	
Yang et al (5)	12		Insufficient patient-level information
Yobbagy et al (38)	1	✓	
Yoshioka et al (39)	1	✓	Yoshioka, Saito, Kawakami, Mineyama, Sekiya (39)
Yung et al (40)	1	✓	

Males composed more than one-half (59%, n = 39) of the study sample. The median age of the patients was 48 years (range, 16-80) (Table 2). Based on imaging, AMH was reported as being unilateral in approximately three-quarters (73%, n = 48) of the patients. Nineteen (40%) of these were in the right adrenal gland and 29 (60%) were in the left. The remaining 18 patients (27%) were reported as having bilateral disease.

**Table 2.** Demographics, location, clinical and biochemical features, radiological and pathological features, genotype, concurrent tumors, treatment, and outcomes of patients with adrenal medullary hyperplasia

Characteristics	N (66)	%	Female (27) 41%	Male (39) 59%	*P*
Median age (range), y	48 (16-80)		53 (19-80)	43 (16-71)	
Unilateral AMH	48	73	23 (85%)	25 (64%)	.06
Right	19/48	40			
Left	29/48	60^b^			
Bilateral AMH	18	27	4 (15%)	14 (36%)	.06
Hypertension (all)	50	76	18 (67%)	32 (82%)	.15
Paroxysmal hypertension	9/50	18	1 (4%)	8 (20%)	.07
Headache	20	30	12 (44%)	8 (21%)	.04^a^
Palpitations	30	45	14 (52%)	16 (41%)	.39
Sweating	22	33	11 (41%)	11 (28%)	.29
Anxiety	10	15	3 (11%)	7 (18%)	.51
Elevated urinary/serum catecholamines	57	86	23 (85%)	34 (87%)	1.00
Presence of MEN2	15	23	6 (22%)	9 (23%)	.98
Presence of other pathological variants	4 (2*NF1*, *MAX*, *SDHB*)	6	1 (*NF1*) (4%)	3 (*SDHB*, *MAX*, *NF1*) (8%)	.64
Concurrent tumors	38	58	16 (59%)	20 (57%)	
Pheochromocytoma	16/38	42	8 (30%)	8 (20%)	.40
MTC	9/38	24	2 (7%)	7 (18%)	.29
Adrenocortical adenoma	11/38	29	5 (18%)	6 (15%)	1.00
Abnormal CT (adrenal enlargement or lesion)	30	46	12 (44%)	18 (27%)	.89
I-MIBG uptake	26	39	9 (33%)	17 (44%)	.40
Histological findings	60^b^	91	26 (96%)	34 (87%)	.39
Adrenalectomy performed	58	88	25 (93%)	33 (85%)	.46
Symptom resolution postadrenalectomy	45/58	78	19 (70%)	26 (67%)	.75

Abbreviations: AMH, adrenal medullary hyperplasia; CT, computed tomography; MEN, multiple endocrine neoplasia; MIBG, meta-iodobenzylguanidine; MTC, medullary thyroid carcinoma.

Urinary catecholamines: includes any of total catecholamine, adrenaline, noradrenaline, dopamine, catecholamine metabolites (metanephrine (total or free), normetanephrine, vanillylmandelic acid) concentrations in the urine.

Serum Catecholamines: includes any of total catecholamine, adrenaline, noradrenaline, dopamine, catecholamine metabolites (metanephrine (total or free), normetanephrine) concentrations in the serum.

Indicates significance on χ^2^ test.

Two of the histological findings were done on autopsy (8, 36).

Specific BP measurements were only reported in one-third (33%, n = 22) of the patients, with “hypertension” being more commonly reported. Using both these methods of identification, hypertension was identified in the majority (76%, n = 50) of patients, with 18% (n = 9/50) being classified as having paroxysmal hypertension. Palpitations were reported by 45% (n = 30) of the patients; sweating by 33% (n = 22); headaches by 30% (n = 20); and anxiety by 15% (n = 10). Other less commonly reported symptoms that led to measurements of catecholamine/metanephrine levels were chest pain, nausea, and vomiting, Cushing-like symptoms, flank pain, and neck swelling. Excess catecholamines were detected in the majority (86%, n = 57) of patients, either in the urine (71%, n = 47) or in the serum (33%, n = 22). These were reported in the form of total catecholamine levels (n = 15), individual catecholamines (n = 31), metanephrines (n = 14), or vanillylmandelic acid (n = 9); in some cases, reported in more than 1 form. Other abnormal hormone levels were also reported in some patients, such as aldosterone (n = 1), 17-ketosteroids (n = 2), 17-hydroxycorticosteroids (n = 2), chromogranin A (n = 1), calcitonin (n = 4), cortisol (n = 5), erythropoietin (n = 1), and ACTH (n = 2).

MEN2 was diagnosed in 23% (n = 15) of the patient group. Other pathological variants were identified in *NF1* (n = 2), *MAX* (n = 1), and *SDHB* (n = 1) genes. Concurrent endocrine tumors were identified with AMH in more than one-half (58%, n = 38) of the patients. The most common of these were pheochromocytoma (42%, n = 16/38) identified mainly in the ipsilateral gland (n = 13), with some in the contralateral gland (n = 2) or both (n = 1), adrenocortical adenoma (29%, n = 11/38), and medullary thyroid carcinoma (MTC) (24%, n = 9/38).

Computed tomography imaging revealed an enlarged adrenal gland or an adrenal tumor in 46% (n = 30) of the patients. Results of alternative imaging modalities included abnormal I-123 MIBG uptake in 39% (n = 26); hypervascularity on adrenal angiography (n = 3); adrenal growth in 3 of the 4 patients who had a magnetic resonance imaging scan; bilateral adrenal involvement in 1 of the 2 patients who had a fluorodopa positron emission tomography scan; a normal ultrasound in most cases (94%, n = 62); and an increased single-photon emission computed tomography uptake in 4 patients. A pressor response was found during an exploratory laparotomy in 2 patients.

Following identification by any of catecholamine excess, imaging abnormalities or pressor response during exploratory laparotomy, 88% (n = 58) of the patients were treated by unilateral or bilateral adrenalectomy, according to tumor location, and the majority (78%, n = 45/58) were described as experiencing postoperative resolution of catecholaminergic symptoms, together with normalization of serum and urinary catecholamine levels. However, 9 patients had persisting low-grade hypertension, managed with medications, and 1 patient died of myocardial infarction after surgery. Apart from hypertension, no other catecholamine associated symptoms were reported. Of the 9 patients who remained hypertensive, incomplete normalization of catecholamine levels was reported in 1 case only. Postsurgical outcomes were not reported in 7 of the patients who underwent adrenalectomy. Long-term follow-up data were not reported for any of the patients with the exception of 1 case in which recurrence of symptoms 8 months postsurgery was attributed possibly to contralateral AMH.

Histological examination of the gland postadrenalectomy showed characteristic abnormalities (thickened medulla with pleomorphic cells and an increase in medulla to cortex ratio >1:10) in the 58 patients who underwent adrenalectomy and in 2 patients who died before having surgery (ie, 91% [n = 60] had histological examination diagnostic of AMH).

### Sex-specific Analysis

Clinical presentation and outcomes were comparable between the sexes (Table 2). The exception was differences in patient-reported headache, which was more common in females than in males (44%, n = 12 vs 21%, n = 8; *P* < .05) (Table 2).

### Age-specific Analysis

There were no differences in reported symptoms and signs between the age groups of patients (Table 3). However, comorbid MTC was more common in the youngest age group (27%, n = 6) than in patients aged 40 to 59 years, in whom there were no cases, or in patients aged 60 years and older (19%, n = 3) (overall *P* < .05 for age group and MTC). In contrast, adrenocortical adenomas were more common in people aged 60 and older (35%, n = 6) compared with those aged 40 to 59 years or those aged 0 to 39 years (15%, n = 4% and 4%, n = 1, respectively; overall *P* < .05 for age group) and comparable to population reports. Adrenalectomy rates also varied by age group. All patients aged 40 to 59 years (n = 27) had an adrenalectomy, compared with 88% (n = 15) of those aged 60 years and older and 73% (n = 16) of those in the youngest group (overall *P* < .05 for age group).

**Table 3.** Demographics, location, clinical and biochemical features, radiological and pathological features, genotype, concurrent tumors, treatment and outcomes of patients with adrenal medullary hyperplasia by age group

Characteristics	Age group, y			*P*
	0-39 (22, 33%)	40-59 (27, 41%)	60+ (17, 26%)	
Unilateral AMH	13 (59%)	21 (78%)	14 (82%)	.22
Bilateral AMH	9 (41%)	6 (22%)	3 (18%)	.22
Hypertension (all)	14 (64%)	23 (85%)	13 (76%)	.22
Paroxysmal HTN	2 (9%)	5 (18%)	2 (12%)	.67
Headache	4 (18%)	12 (44%)	4 (23%)	.11
Palpitation	6 (27%)	16 (59%)	8 (47%)	.08
Sweating	6 (27%)	11 (41%)	5 (29%)	.56
Anxiety	2 (9%)	6 (22%)	2 (12%)	.43
Elevated urinary catecholamines	15 (68%)	21 (78%)	11 (65%)	.62
Elevated serum catecholamines	7 (32%)	10 (37%)	5 (29%)	.86
Elevated urinary/serum catecholamines	18 (82%)	24 (89%)	15 (88%)	.81
Presence of MEN2	7 (32%)	3 (11%)	5 (29%)	.17
Presence of other pathological variants	1 (5%) (*MAX*)	0	3 (18%) (2 *NF1*, 1 *SDHB*)	.06
Concurrent tumors	13 (59%)	11 (39%)	15 (88%)	
Pheochromocytoma	5 (23%)	5 (18%)	6 (35%)	.47
MTC	6 (27%)	0	3 (19%)	.02^a^
Adrenal cortical adenoma	1 (4%)	4 (15%)	6 (35%)	.04^a^
Abnormal CT (adrenal enlargement or lesion)	7 (32%)	14 (58%)	9 (53%)	.29
Adrenalectomy	16 (73%)	27 (100%)	15 (88%)	.01^a^
symptom resolution postadrenalectomy	11 (50%)	21 (78%)	13 (76%)	.08

Abbreviations: AMH, adrenal medullary hyperplasia; CT, computed tomography; MEN, multiple endocrine neoplasia; MTC, medullary thyroid cancer.

Indicates significance on χ^2^ test.

### Unilateral Compared With Bilateral AMH

There were no differences in symptomatology, biochemical findings, or in the incidence of MEN2 and pheochromocytoma/MTC between patients identified as having unilateral or bilateral disease (Table 4). However, more patients (94%, n = 45) with unilateral disease were treated with an adrenalectomy than patients with bilateral disease (72%, n = 13, *P* < .05).

**Table 4.** Demographics, location, clinical and biochemical features, radiological and pathological features, genotype, concurrent tumors, treatment and outcomes of patients with adrenal medullary hyperplasia: comparison of unilateral and bilateral lesion

Characteristics	Unilateral (n = 48)	Bilateral (n = 18)	*P* value
Hypertension	36 (75%)	14 (78%)	1.00
Paroxysmal	6 (12%)	3 (17%)	.70
Headache	14 (29%)	6 (33%)	.74
Palpitation	22 (46%)	8 (44%)	.92
Sweating	17 (35%)	5 (28%)	.56
Anxiety	10 (21%)	0	.05
Elevated urinary catecholamines	35 (73%)	12 (67%)	.62
Elevated serum catecholamines	17 (35%)	5 (28%)	.56
Elevated urinary/serum catecholamines	43 (90%)	14 (78%)	.24
Presence of MEN2	12 (25%)	3 (17%)	.54
Presence of other pathological variants	3 (6%)	1 (6%)	1.00
Pheochromocytoma	14 (29%)	2 (11%)	.20
MTC	6 (12%)	3 (17%)	.70
Adrenocortical adenoma	8 (17%)	3 (17%)	1.00
Abnormal CT (adrenal enlargement or lesion)	22 (46%)	8 (44%)	.92
Adrenalectomy performed	45 (94%)	13 (72%)	.03^a^
Symptom resolution postadrenalectomy	34 (71%)	11 (61%)	.45

Abbreviations: AMH, adrenal medullary hyperplasia; CT, computed tomography; MEN, multiple endocrine neoplasia; MTC, medullary thyroid cancer.

Indicates significance on Chi square test.

### Familial Compared With Sporadic AMH

Hypertension was more common (85%, n = 40) among sporadic than familial cases (53%, n = 10) (*P* < .05). Positive histology was also more common in the sporadic patients (98%, n = 46) than among patients with familial AMH (74%, n = 14) (*P* < .05) (Table 5). Similarly, symptom resolution after adrenalectomy was reported more often among sporadic cases (79%, n = 37) than among those with familial disease (42%, n = 8) (*P* < .05). In contrast, a comorbid diagnosis of pheochromocytoma was more common (63%, n = 12) among familial patients with AMH than among patients with sporadic disease (9%, n = 4) (*P* < .05), as was a comorbid diagnosis of MTC (32%, n = 6 vs 6%, n = 3, respectively; *P* < .05) (Table 5). Of the 12 familial cases of pheochromocytoma, 9 were associated with MEN 2, 2 with *NF1* and 1 with *MAX* pathological variants.

**Table 5.** Demographics, location, clinical and biochemical features, radiological and pathological features, genotype, concurrent tumors, treatment and outcomes of patients with adrenal medullary hyperplasia: comparison of familial and sporadic AMH

Characteristics	Familial (19, 29%)	Sporadic (47, 71%)	P value
Female	7 (37%)	20 (43%)	.67
Male	12 (63%)	27 (57%)	.67
Unilateral AMH	14 (74%)	34 (72%)	.91
Bilateral AMH	5 (26%)	13 (28%)	.91
Hypertension (all)	10 (53%)	40 (85%)	.01^a^
Paroxysmal hypertension	1 (5%)	8 (17%)	.27
Headache	3 (16%)	17 (36%)	.10
Palpitation	7 (37%)	23 (49%)	.37
Sweating	4 (21%)	18 (38%)	.18
Anxiety	5 (26%)	5 (11%)	.14
Elevated urinary/serum catecholamines	18 (95%)	39 (83%)	.27
Pheochromocytoma	12 (63%)	4 (9%)	<.001^a^
MTC	6 (32%)	3 (6%)	.01^a^
Adrenocortical adenoma	2 (11%)	9 (19%)	.49
Abnormal CT (adrenal enlargement or lesion)	8 (42%)	22 (47%)	.73
I-MIBG uptake	6 (32%)	20 (43%)	.41
Histological findings	14 (74%)	46 (98%)	.01^a^
Adrenalectomy performed	17 (89%)	41 (87%)	1.00
Symptom resolution postadrenalectomy	8 (42%)	37 (79%)	.004^a^

Abbreviations: AMH, adrenal medullary hyperplasia; CT, computed tomography; MEN, multiple endocrine neoplasia; MTC, medullary thyroid cancer.

Indicates significance on χ^2^ test.

## Discussion

This study is the largest and most comprehensive examination of reported cases of AMH to date. It analyzed data from 66 patients extracted from 29 articles. Of the 66 patients in the study group, the majority were identified as having unilateral disease, more commonly in the left adrenal gland, although bilateral AMH could not be excluded in patients not having an adrenalectomy. All patients had signs and symptoms of catecholamine excess, with hypertension being the most common. Elevated catecholamine levels, in either urine or serum, were detected in most patients. Most cases of AMH were identified as being sporadic. Among the familial cases, MEN2 was the most common genetic disorder, affecting 23% of the sample, with a further 4 cases associated with *NF1*, *MAX*, and *SDHB* pathological variants. More than one-half of the study sample had concurrent endocrine tumors, most commonly pheochromocytoma, MTC, or an adrenocortical adenoma. Abnormalities on imaging, with computed tomography and MIBG scanning being the most frequently used imaging modalities, were detected in around 80% of the sample suggesting that imaging abnormalities are likely to form the basis of an AMH diagnosis in the majority of patients. Adrenalectomy is an effective treatment for most patients, with the majority achieving resolution of symptoms and catecholamine excess. Histological confirmation of AMH was reported in almost all patients.

There were few differences in presentation, symptoms, and outcomes between the sexes. Although not statistically significant, bilateral disease was more common in males than females and, although there is evidence to suggest that bilateral disease may be more common in patients with MEN2 (11), this was not found among the patients in this sample. Of all the symptoms and signs associated with AMH, only headache, which was present in approximately one-third of this sample, appeared to differ in frequency between the sexes, being twice as common in females than males.

Although the median age of patients was 48 years, the sample included teenagers and the elderly, suggesting broad adult incidence of AMH. AMH-related symptoms and signs were not age-related. However, a concurrent MTC at the time of AMH diagnosis was more common among younger patients. Despite the association between MTC and the MEN2 and the advice that testing for *RET* proto-oncogene variants is recommended, particularly in young patients, an association between age and a MEN2 diagnosis was not evident, possibly because of a lack of access to genetic screening in some countries and the small size of this sample of reported AMH patients (41, 42). Adrenal adenomas are a common condition among people aged 60 years or older (43); therefore, it is more likely that patients in this age group may have an adrenalectomy for a suspected adenoma but be found to have an incidental diagnosis of AMH. However, in this analysis AMH patients aged older than 60 only composed one-quarter of the study cohort.

Most patients were treated with an adrenalectomy, either unilateral or bilateral, with successful postoperative symptom resolution in the majority. These results support the findings of a previous study which recommended adrenalectomy as the most effective treatment for AMH (5). However, the results of this analysis also highlight the problem of persisting symptoms postsurgery in a minority of patients. In this sample, resolution of preoperative symptoms was common in those older than age 40 years, with surgery appearing to be least effective in the youngest patients. The reasons for this are uncertain but may be due to the presence of unidentified and, therefore, undiagnosed AMH in the contralateral adrenal gland which highlights the importance of multidisciplinary discussions in evaluating patients before and after surgery to plan tailored treatment and follow-up (43).

Patients with bilateral AMH reported similar symptoms and had comparable levels of biochemical abnormalities to those with unilateral disease. Adrenalectomy, however, was more frequently performed in patients with unilateral rather than bilateral disease. The reasons for this are unknown, but may reflect a desire to avoid bilateral adrenalectomy and the inevitable postoperative diagnosis of adrenal insufficiency, with its requirement for lifelong glucocorticoid replacement therapy and its attendant risks of complications from glucocorticoid exposure and adrenal crises (5, 44). Details on the type of surgery used were not available, but evidence indicates that bilateral partial adrenalectomy may be the preferred option for patients with bilateral disease (5). This procedure has been demonstrated previously in 2 cases of AMH, 1 in which partial bilateral adrenalectomy was undertaken at 1 operation and another in which unilateral adrenalectomy did not lead to symptom resolution and was followed by partial adrenalectomy on the contralateral side. In both cases, bilateral partial adrenalectomy resulted in reduced AMH symptoms and the consequent likelihood of AMH-related complications, while at the same time preserving adrenocortical function (5).

The concurrence of pheochromocytoma and MTC among the familial AMH cases supports a genetic association between familial pheochromocytoma and MTC with *RET*, *SDHD*, *MAX*, and *NF1* pathological variants being reported (45). For this reason, genetic screening in these patients is strongly recommended. Diagnoses of familial pheochromocytoma and AMH associated with these pathological variants are often identified as bilateral (45), possibly explaining the lower rates of symptom resolution in familial compared with sporadic AMH. Histological confirmation was more common in sporadic than familial AMH, possibly reflecting access to confirmation of AMH indirectly via genetic screening in some patients with familial AMH. However, some of the histologically confirmed cases of AMH were detected incidentally during investigation for an adrenal mass.

In this study, we conducted an extensive literature search and included all cases of AMH published to date, assessing patterns of presentation and outcomes in patient subgroups. Because AMH is very rare, our sample was limited in scope and comprised retrospective data from case reports and case series that were not always complete or comprehensive. Where symptoms or signs were not reported, the assumption was made that the relevant factor was not present, which may have resulted in an underestimate of the prevalence of some symptoms. In addition, 29% of the included cases were from 1 institution (4), which may have introduced some bias into the study.

In conclusion, AMH is a rare disorder with limited literature available to support clinical decision-making. This study demonstrated that AMH is more often sporadic; associated with MEN2 in about one-quarter of patients; more often diagnosed as unilateral; associated with 1 or more symptoms of catecholamine excess; and often detectable on imaging. Adrenalectomy was the most common method of treatment with most patients achieving cure postsurgery. Future analyses of larger samples should enable improved delineation of patterns of presentation and better predictors of health outcomes that could assist in the clinical management of patients.

## Data Availability

Original data generated and analyzed during this study are included in this published article.

## Abbreviations

AMH: adrenal medullary hyperplasia
BP: blood pressure
MEN: multiple endocrine neoplasia
MIBG: meta-iodobenzylguanidine
MTC: medullary thyroid cancer
